# Segmental transverse colectomy. Minimally invasive versus open approach: results from a multicenter collaborative study

**DOI:** 10.1007/s13304-021-01159-4

**Published:** 2021-09-14

**Authors:** Marco Milone, Maurizio Degiuli, Nunzio Velotti, Michele Manigrasso, Sara Vertaldi, Domenico D’Ugo, Giovanni Domenico De Palma, and Italian Society of Surgical Oncology Colorectal Cancer Network (SICO CCN) group, Marco Ettore Allaix, Carlo Alberto Ammirati, Gabriele Anania, Andrea Barberis, Andrea Belli, Francesco Bianco, Paolo Pietro Bianchi, Cristina Bombardini, Dario Bruzzese, Davide Cavaliere, Claudio Coco, Andrea Coratti, Giovanni De Manzoni, Paola De Nardi, Giuseppe De Simone, Raffaele De Luca, Paolo Delrio, Antonio Di Cataldo, Katia Di Lauro, Alberto Di Leo, Annibale Donini, Ugo Elmore, Andrea Fontana, Giampaolo Formisano, Sergio Gentilli, Giuseppe Giuliani, Luigina Graziosi, Mario Guerrieri, Giovanni Li Destri, Roberta Longhin, Michela Mineccia, Manuela Monni, Mario Morino, Monica Ortenzi, Ugo Pace, Francesca Pecchini, Corrado Pedrazzani, Micaela Piccoli, Sara Pollesel, Salvatore Pucciarelli, Rossella Reddavid, Daniela Rega, Marco Rigamonti, Gianluca Rizzo, Riccardo Rosati, Franco Roviello, Mauro Santarelli, Federica Saraceno, Stefano Scabini, Giuseppe Servillo, Giuseppe Sigismondo Sica, Pierpaolo Sileri, Michele Simone, Luigi Siragusa, Silvia Sofia, Leonardo Solaini, Angela Tribuzi, Giulia Turri, Andrea Vignali, Matteo Zuin, Michele Zuolo

**Affiliations:** 1 Department of Clinical Medicine and Surgery, Federico II University of Naples, Via Sergio Pansini, 5, 80131 Naples, Italy; 2 Department of Oncology, Surgical Oncology and Digestive Surgery Unit, San Luigi University Hospital, Orbassano, Turin, Italy; 3 Department of Advanced Biomedical Sciences, Federico II University of Naples, Naples, Italy; 4 Department of General Surgery, Sacred Heart Catholic University, Rome, Italy

**Keywords:** Minimally invasive surgery, Transverse colon cancer, Laparoscopic, Robotic

## Abstract

The role of minimally invasive surgery in the treatment of transverse colon cancer is still controversial. The aim of this study is to investigate the advantages of a totally laparoscopic technique comparing open versus laparoscopic/robotic approach. Three hundred and eighty-eight patients with transverse colon cancer, treated with a segmental colon resection, were retrospectively analyzed. Demographic data, tumor stage, operative time, intraoperative complications, number of harvested lymph nodes and recovery outcomes were recorded. Recurrences and death were also evaluated during the follow-up. No differences were found between conventional and minimally invasive surgery, both for oncological long-term outcomes (recurrence rate *p *= 0.28; mortality *p *= 0.62) and postoperative complications (overall rate *p *= 0.43; anemia *p *= 0.78; nausea *p *= 0.68; infections *p *= 0.91; bleeding *p *= 0.62; anastomotic leak *p *= 0.55; ileus *p *= 0.75). Nevertheless, recovery outcomes showed statistically significant differences in favor of minimally invasive surgery in terms of time to first flatus (*p *= 0.001), tolerance to solid diet (*p *= 0.017), time to first mobilization (*p *= 0.001) and hospital stay (*p *= 0.004). Compared with laparoscopic approach, robotic surgery showed significantly better results for time to first flatus (*p *= 0.001), to first mobilization (*p *= 0.005) and tolerance to solid diet (*p *= 0.001). Finally, anastomosis evaluation confirmed the superiority of intracorporeal approach which showed significantly better results for time to first flatus (*p *= 0.001), to first mobilization (*p *= 0.003) and tolerance to solid diet (*p *= 0.001); moreover, we recorded a statistical difference in favor of intracorporeal approach for infection rate (*p *= 0.04), bleeding (*p *= 0.001) and anastomotic leak (*p *= 0.03). Minimally invasive approach is safe and effective as the conventional open surgery, with comparable oncological results but not negligible advantages in terms of recovery outcomes. Moreover, we demonstrated that robotic approach may be considered a valid option and an intracorporeal anastomosis should always be preferred.

## Introduction

Laparoscopic resection for colorectal cancer has already demonstrated long- and short-term benefits both for recovery outcomes and oncological safety [1]. However, the role of minimally invasive surgery in the treatment of transverse colon cancer is still controversial: the majority of previous randomized trials have excluded transverse colon cancer because the completeness of resection requires a technically difficult lymph node dissection around the middle colic artery and a hard reconstruction of intestinal continuity [2]. Over the last years, the increasing experience in laparoscopic and robotic colonic resections among surgeons has led to the cumulative publication of several studies comparing the oncological outcomes of the open to the minimally invasive approach for transverse colon cancer, but the real “gold standard” is still far to be defined [3, 4].

The aim of the study is to investigate the short- and long-term outcomes of transverse colon cancer surgery comparing open versus laparoscopic/robotic approach and a subgroup analysis will be performed to compare laparoscopic vs robotic resection and intracorporeal vs extracorporeal anastomosis.

## Materials and methods

### Study population and design

Three hundred and eighty-eight consecutive patients with mid transverse colon cancer, treated with a segmental colon resection between 2006 and 2016 in 28 Italian high-volume (more than 70 procedures/year) centers were retrospectively analyzed for this study. The protocol for research was approved by the institutional review board of all participating centers.

Mid transverse colon cancer was defined, after surgical exploration, as a tumor located in the mid part of the transverse colon excluding the 10 cm distal third in the left colonic angle (splenic flexure) and the 10 cm proximal part in the right colonic angle (hepatic flexure) of the transverse colon cancer [5].

All patients were operated by expert surgeons (more than 50 procedures/year) with open or minimally invasive (laparoscopic and robotic) approach.

To minimize the bias related to the different surgical techniques, only procedures performed according to the standardized criteria were included in the study: transverse colectomy is defined as the resection of a variable length of bowel included between the hepatic and the splenic flexure, with its lymph vascular supply along the middle colic pedicle, whose ligation is done at its origin. Both flexures are mobilized and the continuity of the bowel restored by fashioning an end-to-end or side-to-side anastomosis. In case of minimally invasive approach, the anastomosis was performed with intra- or extracorporeal technique depending on the advice of the surgeon. The period between surgery and discharge in all enrolled patients followed ERAS perioperative care protocol [6].

### Outcomes

Primary outcomes: analyses comparing open versus minimally invasive approach in segmental transverse colectomy were performed. Moreover, between patients who underwent minimally invasive techniques, we compared outcomes from laparoscopic versus robotic approach.

Secondary outcomes: an intra- and extracorporeal anastomosis technique in the minimal invasive surgery was compared in the study population.

### Data assessment

Demographic data (gender, age, body mass index (BMI), ASA), tumor stage (TNM), operative time, intraoperative complications, number of lymph nodes harvested, time to first flatus, mobilization, tolerance to solid diet and hospital discharge, were recorded.

We also collected data about postoperative surgical complications according to Clavien–Dindo classification [7]: surgical wound infections, anastomotic leakage, prolonged ileus and abdominal or bowel bleeding. The anastomotic leakage was defined as a condition of clinical or radiological anastomotic dehiscence that needed or did not need surgical revision. All centers performed a complete blood examination panel daily including C-reactive protein evaluation for subclinical leaks until patient discharge. We considered as bleeding the cases that required blood transfusion; on the other hand, we considered as anemia cases in which patients had a decrease in hemoglobin that did not need blood transfusions.

Pathological outcomes included the specimen length, the distance of the tumor from the proximal and the distal margin all measured in centimeters. We also reported the number of lymph nodes harvested and how many of these contained metastasis. Recurrences and death were also evaluated during the follow-up.

### Statistical analysis

The analysis was performed with SPSS version 20.0 (IBM, Armonk, NY).

Continuous variables are described as median and interquartile range and compared by the Mann–Whitney *U* test. Categorical variables are reported as percentages and compared by the Fisher’s exact test. A *p* value of less than 0.05 was defined as statistically significant.

Propensity scores were obtained by a logistic regression model. The surgical technique (laparoscopic vs robotic) and the anastomosis technique (intra vs extracorporeal) were entered into the regression model as the dependent variable, and baseline patient and tumor characteristics (age, sex, BMI, ASA score and TNM stage) were the independent variables. Matching of the propensity scores was obtained with the “1:1 nearest neighbor” matching method, with a conservative caliper width of 20% of the standard deviation of the log of propensity score.

## Results

A total of 388 patients with mid transverse colon cancer who underwent segmental resection were included in the study. Of these, 224 (57.7%) patients underwent open surgery and 164 (42.3%) underwent a minimally invasive approach; particularly, 146 (89%) had a laparoscopic segmental colon resection and 18 (11%) had a robotic colon resection. In terms of post-resection anastomosis, 33 (22.6%) patients received an intracorporeal anastomosis and 131 (77.4%) patients had an extracorporeal approach.

### Primary outcomes

#### Open vs minimally invasive surgery

Firstly, we compared the characteristics of patients who underwent open surgery versus patients who underwent a minimally invasive approach. The two treatment groups were entirely matched for the analyzed characteristics; thus, no propensity matching was needed for further analysis. (Table 1).

**Table 1:** Comparison between conventional open approach and minimally invasive surgery

	Open (*n* = 224)	Minimally (*n* = 164)	*p*
Males (*n*, %)	120 (53.57%)	72 (43.9%)	0.2
Age (median, IQR)	72 (22.5)	72 (21.5)	0.1
BMI (median, IQR)	19 (21)	19 (19)	0.1
ASA score (median, IQR)	2 (1)	2 (1)	0.5
T (median, IQR)	3 (0)	3 (1)	0.1
N (median, IQR)	0 (1)	0 (1)	0.8
M (median, IQR)	0 (0)	0 (0)	0.3
Complications (*n*, %)	71 (31.7%)	46 (28%)	0.43
Anemia (*n*, %)	5 (2.2%)	3 (1.8%)	0.78
Nausea (*n*, %)	7 (3.1%)	4 (2.4%)	0.68
Infections (*n*, %)	10 (4.4%)	7 (4.2%)	0.91
Bleeding (n, %)	12 (5.3%)	7 (4.2%)	0.62
Leakage (*n*, %)	11 (4.9%)	6 (3.6%)	0.55
Ileus (*n*, %)	2 (0.9%)	1 (0.6%)	0.75
Recurrences (*n*, %)	51 (22.8%)	30 (18.3%)	0.28
Death (*n*, %)	15 (6.7%)	9 (5.5%)	0.62
Operative time (median, IQR)	157 (80)	140 (75)	0.102
Clavien (median, IQR)	0 (1)	0 (1)	0.036
Time to first flatus (median, IQR)	4 (2)	3 (2)	0.001
Solid diet (median, IQR)	4 (3)	4 (2)	0.017
Mobilization (median, IQR)	2 (1)	1 (1)	0.001
Hospital stay (median, IQR)	9 (5)	7.5 (4)	0.004
Lymph nodes + (median, IQR)	0 (1)	0 (1)	0.19
Total lymph nodes (median, IQR)	13 (8)	15 (7)	0.33
Specimen length (median, IQR)	20 (11.6)	20 (12)	0.65
Proximal margin (median, IQR)	7 (7)	7 (5)	0.46
Distal margin (median, IQR)	8 (7)	10 (6.5)	0.14

*IQR* interquartile range, *BI*: body mass index, *ASA* American Society of Anesthesiologists

There were no differences in demographic characteristics such as sex (53.57% vs 43.9% males; *p *= 0.2), age (72 median, 22.5 IQR vs 72 median, 21.5 IQR; *p *= 0.1), BMI (19 median, 21 IQR vs 19 median, 19 IQR; *p *= 0.1) and ASA score (2 median, 1 IQR vs 2 median, 1 IQR; *p *= 0.5). The two groups were also homogeneous for tumor stage characteristics: T (3 median, 0 IQR vs 3 median, 1 IQR; *p *= 0.1)—N (0 median, 1 IQR vs 0 median, 1 IQR; *p *= 0.8)—M (0 median, 0 IQR vs 0 median, 0 IQR *p *= 0.3).

Median operative time was 157 (80 IQR) min for open resections and 140 (75 IQR) min for minimally invasive approach (*p *= 0.102).

Recovery outcomes showed statistically significant differences in favor of minimally invasive surgery in terms of time to first flatus (4 median, 2 IQR vs 3 median, 2 IQR days; *p *= 0.001), tolerance to solid diet (4 median, 3 IQR vs 4 median, 2 IQR days; *p *= 0.017), time to first mobilization (2 median, 1 IQR vs 1 median, 1 IQR days; *p *= 0.001) and hospital stay (9 median, 5 IQR vs 7.5 median, 4 IQR days; *p *= 0.004).

About complications, we found no statistical differences: overall rate (31.7% vs 28%; *p *= 0.43), anemia (2.2% vs 1.8%; *p *= 0.78), nausea (3.1% vs 2.4%; *p *= 0.68), infections (4.4% vs 4.2%; *p *= 0.91), bleeding (5.3% vs 4.2%; *p *= 0.62), anastomotic leak (4.9% vs 3.6%; *p *= 0.55) and ileus (0.9% vs 0.6%; *p *= 0.75) were reported to be comparable between groups.

In terms of pathological outcomes, we found no differences in specimen length (20 median, 11.6 IQR vs 20 median, 12 IQR; *p *= 0.65), proximal margin (7 median, 7 IQR vs 7 median, 5 IQR; *p *= 0.46) and distal margin (8 median, 7 IQR vs 10 median, 6.5 IQR; *p *= 0.14), number of total lymph nodes harvested (13 median, 8 IQR vs 15 median, 7 IQR; *p *= 0.33) and number of lymph nodes positive for metastasis (0 median, 1 IQR vs 0 median, 1 IQR; *p *= 0.19). Finally, the mean follow-up of 3.4 ± 2.1 years for open approach and 3.3 ± 2.3 years for minimally invasive approach showed no differences between the two groups both in terms of recurrence rate (22.8% vs 18.3%; *p *= 0.28) and mortality (6.7% vs 5.5%; *p *= 0.62).

#### Laparoscopic vs robotic approach

We compared 146 patients who underwent laparoscopic surgery versus 18 patients underwent robotic surgery: the two groups were not homogeneous for baseline characteristics, so propensity score match was applied, resulting in a study population of 36 patients: 18 for each group (Table 2a, b).

**Table 2:** (a) Pre- and post-matching data between laparoscopic and robotic group (b) comparison between laparoscopic and robotic approach

a			
Pre-matching	Laparoscopic (*n *= 146)	Robotic (*n* = 18)	*p*
Males (*n*, %)	57 (39%)	15 (83.3%)	0.001
Age (median, IQR)	73.5 (4)	74.2 (6.7)	0.04
BMI (median, IQR)	25 (6)	25 (10.5)	0.001
ASA score (median, IQR)	2 (1)	3 (1)	0.001
T (median, IQR)	3 (1)	3 (0)	0.001
N (median, IQR)	0 (1)	0.5 (1)	0.03
M (median, IQR)	0 (0)	0 (0)	0.001

Post-matching	Laparoscopic (*n* = 18)	Robotic (*n* = 18)	*p*
Males (*n*, %)	16 (88.8%)	15 (83.3%)	0.99
Age (median, IQR)	73.2 (5.7)	74.2 (6.7)	0.82
BMI (median, IQR)	26.6 (5.6)	25 (10.5)	0.7
ASA score (median, IQR)	2 (0)	3 (1)	0.43
T (median, IQR)	2 (2)	3 (0)	0.78
N (median, IQR)	0 (0)	0.5 (1)	0.48
M (median, IQR)	0 (0)	0 (0)	0.59

b			
	Laparoscopic (*n *= 18)	Robotic (*n *= 18)	*p*
Complications (*n*, %)	7 (28.8%)	4 (22.2%)	0.47
Anemia (*n*, %)	2 (11.1%)	1 (5.5%)	0.91
Nausea (*n*, %)	3 (16.6%)	1 (5.6%)	0.6
Infections (*n*, %)	5 (27.7%)	2 (11.1%)	0.4
Bleeding (*n*, %)	5 (27.7%)	2 (11.1%)	0.4
Leakage (*n*, %)	4 (22.2%)	2 (11.1%)	0.65
Ileus (*n*, %)	1 (5.5%)	0	0.98
Recurrences (*n*, %)	6 (33.3%)	4 (22.2%)	0.71
Death (*n*, %)	7 (38.8%)	2 (11.1%)	0.12
Operative time (median, IQR)	148.5 (51.2)	157.5 (60)	0.82
Clavien (median, IQR)	0 (0.7)	0 (0)	0.266
Time to first flatus (median, IQR)	3.5 (1.7)	2 (0.7)	0.001
Solid diet (median, IQR)	4.5 (1.7)	2 (0)	0.001
Mobilization (median, IQR)	1.5 (1)	1 (0)	0.005
Hospital stay (median, IQR)	8 (4)	8 (2)	0.33
Lymph nodes + (median, IQR)	0 (0.7)	0 (1)	0.276
Total lymph nodes (median, IQR)	11.5 (9.5)	14.5 (3.7)	0.774
Specimen length (median, IQR)	20 (6.2)	30 (3)	0.47
Proximal margin (median, IQR)	7.5 (2)	8 (9)	0.199
Distal margin (median, IQR)	9.7 (5.2)	18 (8)	0.42

*IQR* interquartile range, *BMI* body mass index, *ASA* American Society of Anesthesiologists

Post-matching no differences were found in sex (88.8% vs 83.3,5% males; *p *= 0.99), age (73.2 median, 5.7 IQR vs 74.2 median, 6.7 IQR; *p *= 0.82), BMI (26.6 median, 5.6 IQR vs 25 median, 10.5 IQR kg/m^2^; *p *= 0.7) and ASA score (2 median, 0 IQR vs 3 median, 1 IQR; *p *= 0.43). About tumor staging, we recorded no differences between groups [T: 2 (2 IQR) vs 3 (0 IQR) *p *= 0.78; N: 0 (0 IQR) vs 0.5 (1 IQR) *p *= 0.48; M: 0 (0 IQR) vs 0 (0 IQR) *p *= 0.59].

Also, operative time was comparable between the two approaches [148.5 (51.2 IQR) vs 157.5 (60 IQR) min *p *= 0.82].

In terms of recovery, robotic approach showed significantly better results for time to first flatus (3.5 median, 1.7 IQR vs 2 median, 0.7 IQR days; *p *= 0.001), to first mobilization (1.5 median, 1 IQR vs 1 median, 0 IQR days; *p *= 0.005) and tolerance to solid diet (4.5 median, 1.7 IQR vs 2 median, 0 IQR days; *p *= 0.001); no differences were recorded in hospital stay (8 median, 4 IQR vs 8 median, 2 IQR days; *p *= 0.33).

About complications, we recorded no statistical differences between the two approaches for all examined outcomes: overall rate (28.8% vs 22.2%; *p *= 0.47), anemia (11.1% vs 5.5%; *p *= 0.91), nausea (16.6% vs 5.6%; *p *= 0.6), infections (27.7% vs 11.1%; *p *= 0.4), bleeding (27.7% vs 11.1%; *p *= 0.4), anastomotic leak (22.2% vs 11.1%; *p *= 0.65) and ileus (5.5% vs 0%; *p *= 0.98).

Analysis of pathological outcomes showed no differences in the number of lymph nodes harvested (11.5 median, 9.5 IQR vs 14.5 median, 3.7 IQR; *p *= 0.77), lymph nodes positive for metastasis (0 median, 0.7 IQR vs 0 median, 1 IQR; *p *= 0.37), proximal margin (7.5 median, 2 IQR vs 8 median, 9 IQR; *p *= 0.19), distal margin (9.7 median, 5.2 IQR vs 18 median, 8 IQR; *p *= 0.42) and specimen length (20 median, 6.2 IQR vs 30 median, 3 IQR; *p *= 0.47).

At the mean follow-up of 3.6 ± 2.4 years for laparoscopic and 3.4 ± 2.2 years for robotic approach, data showed no differences between the two groups both in terms of recurrence rate (33.3% vs 22.2%; *p *= 0.71) and mortality (38.8% vs 11.1%; *p *= 0.12).

### Secondary outcome

#### Intra- versus extracorporeal anastomosis

Overall, 33 patients who underwent intracorporeal anastomosis were compared with 131 patients who underwent extracorporeal anastomosis: the two groups showed significant differences in demographic characteristics, so these inhomogeneities required propensity score match analysis (Table 3a, b).

**Table 3:** (a) Pre- and post-matching data between intracorporeal and extracorporeal anastomosis (b) comparison between intracorporeal and extracorporeal anastomosis

a			
Pre-matching	Intracorporeal (*n* = 33)	Extracorporeal (*n* = 131)	*p*
Males (*n*, %)	18 (54.54%)	54 (41.22%)	0.16
Age (median, IQR)	74 (12)	67 (10.6)	0.01
BMI (median, IQR)	25.6 (6.4)	21.5 (10.7)	0.001
ASA score (median, IQR)	2.5 (1.1)	2.1 (0.6)	0.001
T (median, IQR)	3 (1)	3 (1)	0.04
N (median, IQR)	0 (1)	0 (0)	0.01
M (median, IQR)	0 (0)	0 (0)	0.001

Post-matching	Intracorporeal (*n* = 33)	Extracorporeal (*n* = 33)	*p*
Males (*n*, %)	18 (54.54%)	15 (45.45%)	0.54
Age (median, IQR)	74 (12)	69 (13)	0.21
BMI (median, IQR)	25.6 (6.4)	22.7 (5.7)	0.47
ASA score (median, IQR)	2.5 (1.1)	3 (1)	0.33
T (median, IQR)	3 (1)	3 (1)	0.77
N (median, IQR)	0 (1)	0 (1)	0.12
M (median, IQR)	0 (0)	0 (0)	0.56

b			
	Intracorporeal (*n* = 33)	Extracorporeal (*n* = 33)	*p*
Complications (*n*, %)	4 (12.1%)	15 (48.5%)	0.001
Anemia (*n*, %)	1 (3%)	2 (1.5%)	0.55
Nausea (*n*, %)	1 (3%)	2 (1.5%)	0.55
Infections (*n*, %)	1 (3%)	6 (18.1%)	0.04
Bleeding (*n*, %)	1 (3%)	6 (18.1%)	0.04
Leakage (*n*, %)	0	6 (18.1%)	0.03
Ileus (*n*, %)	0	1 (3%)	0.314
Recurrences (*n*, %)	6 (18.2%)	6 (18.2%)	0.99
Death (*n*, %)	0	2 (6.1%)	0.151
Operative time (median, IQR)	160 (105)	185 (70)	0.005
Clavien (median, IQR)	0 (0)	0 (1)	0.266
Time to first flatus (median, IQR)	3 (1)	4 (1)	0.001
Solid diet (median, IQR)	4 (1)	5 (1)	0.001
Mobilization (median, IQR)	1 (1)	2 (1)	0.003
Hospital stay (median, IQR)	7 (3)	8 (4)	0.564
Lymph nodes + (median, IQR)	0 (2)	0 (0)	0.184
Total lymph nodes (median, IQR)	11 (8)	11 (9)	0.612
Specimen length (median, IQR)	21 (9)	19 (7)	0.136
Proximal margin (median, IQR)	8 (4)	7 (4)	0.432
Distal margin (median, IQR)	10 (8)	8 (5)	0.623

*IQR* interquartile range, *BMI* body mass index, *ASA* American Society of Anesthesiologists

Post-matching, the study population was composed of 66 patients (33 for each group) and there were no significant differences between the groups with regard to the baseline demographic variables [sex: 54.5% vs 45.45% males, *p *= 0.54; age: 74 (12 IQR) vs 69 (13 IQR) years, *p *= 0.21; BMI: 25.6 (6.4 IQR) vs 22.7 (5.7 IQR) kg/m^2^, *p *= 0.47; ASA: 2.5 (1.1 IQR) vs 3 (1 IQR), *p *= 0.33] as well as the stage of the tumors [T: 3 (1 IQR) vs 3 (1 IQR), *p *= 0.77; N: 0 (1 IQR) vs 0 (1 IQR), *p *= 0.12; M: 0 (0 IQR) vs 0 (0 IQR), *p *= 0.56).

Operative time was significantly shorter for intracorporeal anastomosis (160 median, 105 IQR vs 185 median, 70 IQR mins; *p *= 0.005).

Recovery outcomes showed that intracorporeal anastomosis had significantly better results for time to first flatus (3 median, 1 IQR vs 4 median, 1 IQR days; *p *= 0.001), to first mobilization (1 median, 1 IQR vs 2 median, 1 IQR days; *p *= 0.003) and tolerance to solid diet (4 median, 1 IQR vs 5 median, 1 IQR; *p *= 0.001); no differences were recorded in hospital stay (7 median, 3 IQR vs 8 median, 4 IQR days; *p *= 0.56).

In terms of complications, we recorded a statistical difference in favor of intracorporeal approach for overall rate (12.1% vs 48.5%; *p *= 0.001), infections (6.1% vs 24.2%; *p *= 0.04), bleeding (6.1% vs 39.4%; *p *= 0.001) and anastomotic leak (3% vs 18.1%; *p *= 0.03).

Analysis of pathological outcomes showed no differences in the number of lymph nodes harvested (11 median, 8 IQR vs 11 median, 9 IQR; *p *= 0.61), lymph nodes positive for metastasis (0 median, 2 IQR vs 0 median, 0 IQR; *p *= 0.18), proximal margin (8 median, 4 IQR vs 7 median, 4 IQR; *p *= 0.43), distal margin (10 median, 8 IQR vs 8 median, 5 IQR; *p *= 0.62) and specimen length (21 median, 9 IQR vs 19 median, 7 IQR; *p *= 0.13).

Data from a mean follow-up of 3.1 ± 2.2 years for intracorporeal and 3.2 ± 2.3 years for extracorporeal anastomosis again showed the same results for the two groups, both in terms of recurrence rate (18.2% vs 18.2%; *p *= 0.99) and mortality (0% vs 6.1%; *p *= 0.15).

## Discussion

Recent RCTs and several reviews have demonstrated that minimally invasive surgery is nowadays considered the gold standard approach for colorectal cancers with excellent short-term outcomes and equivalent long-term oncologic outcomes compared to conventional open surgery [8–10]. However, less is known about minimally invasive approach to transverse colon cancer, both for its relatively low incidence and for its technical difficulties, which are higher than that for other site colon cancers: the challenging localization of the neoplasm, isolation and ligation of the middle colic vessels, lymphadenectomy, anatomical features of the transverse colon and its relationships with the spleen, pancreas, superior mesenteric vein, duodenum and Treitz ligament make transverse colon mobilization and dissection a challenging procedure.

Cumulating surgeons experience in this kind of surgical approach resulted in several studies which have compared minimally invasive and open surgery for transverse colon cancer [11–13]; however, the interpretation of the data is still limited. The major limitation is the complete absence in literature of studies which analyze and compare the open versus laparoscopic approach to transverse colon cancer on a specific type of resection: in fact, we can only find conclusions based on the mixed results from extended and segmental resections.

We have for the first time, in our best knowledge, compared open versus laparoscopic surgery for segmental resection of transverse colon cancer.

Since knowledge is lacking about the real surgical “gold standard” for the treatment of transverse colon cancer, the decision whether to perform an extended colectomy or a segmental transverse colectomy is based on a surgeon’s preference and segmental resection is one of the possibilities to treat this tumor location [14–16].

A recent systematic review with meta-analysis by Milone et al. [17] which involved 11,687 patients found that there were no statistical differences between transverse colectomy and extended hemicolectomy in short- and long-term oncological outcomes, concluding that the current literature gives the opportunity to perform either a segmental resection or an extended hemicolectomy to treat transverse colon cancer. In our study, we evaluated the advantages of a totally minimally invasive approach to transverse colon cancer and comparing it to the outcomes of open surgery. Evaluating the differences between conventional and minimally invasive surgery, our findings demonstrated that both approaches have similar oncological long-term outcomes without discrepancies in terms of postoperative complications. Nevertheless, in line with current literature [4, 18], minimally invasive techniques revealed better recovery results: return of bowel function and resumption of solid diet occurred significantly earlier in the minimally invasive group with a significantly shorter length of hospital stay. Thus, on the basis of our results, we can confirm that the minimally invasive approach to transverse colon cancer is feasible and safe, with similar operative time, morbidity and mortality rates when compared to the open approach.

In the area of mini-invasive surgery, we refined our analysis comparing patients who had undergone laparoscopic resection with those undergoing robotic resection. The current literature is very poor about the possibility of performing a transverse colon resection with a robotic approach but, because of high technical demand of this procedure, the advantages of robotic surgery, such as stable three-dimensional magnified view, can be maximized. de Angelis et al. [19] described their first experience on 22 patients who underwent robotic transverse colectomy matched with 22 patients who received a laparoscopic resection. The authors found no group differences in complications, recovery and oncological outcomes except for conversion rate which was higher in the laparoscopic population. Similarly, Jung and colleagues [20] described three robotic transverse colectomies, using a hand-sewn intracorporeal anastomosis; the authors found encouraging oncological outcomes with a low rate of complications and concluded that robotic transverse colectomy seems to be a safe and feasible technique which may minimize the necessity of mobilizing both colonic flexures, with facilitated intracorporeal anastomosis.

In our results, the analysis between patients who underwent laparoscopic or robotic surgery demonstrated equivalence of the two approaches except for the time to first flatus, tolerance to solid diet and patients’ mobilization which were lower in case of robotic procedures.

Finally, anastomosis evaluation confirmed that intracorporeal approach shows some advantages in terms of complications and recovery outcomes. As previously demonstrated [21], this can be easily explained because an intracorporeal end-to-end anastomosis allows to avoid an excessive dissection and colonic flexure takedown to extract the specimen; on the other hand, an extracorporeal anastomosis requires a mobilization of transverse colonic mesentery and of both colonic flexure to reach the mini-laparotomy site [22, 23]. van Oostendorp and colleagues [24] recently furnished a systematic review on 1492 patients who underwent laparoscopic right colectomy: they found a larger decrease in short-term morbidity and a significant decrease in the length of stay in favor of intracorporeal anastomosis. Similarly, Kelly et al.[25] analyzed data from 21 consecutive patients with a right colon cancer who underwent robotic resection with an intracorporeal anastomosis. The authors observed a low complication rate with good oncological and recovery outcomes, concluding that an intracorporeal approach is safe and feasible. Despite these studies, current literature lacks data about differences and advantages of intra- and extracorporeal anastomosis in transverse colon resections; our results, by this point of view, provide support to the effectiveness of intracorporeal anastomotic technique to provide surgeons a clearer perspective on which to perform a conscious choice during this type of colic resection.

## Conclusion

Our results confirm that minimally invasive approach, both laparoscopic and robotic, is as safe and effective in transverse colon surgery as the conventional open surgery, with comparable oncological results and not negligible advantages in terms of recovery outcomes. Moreover, our analysis demonstrated that fashioning intracorporeal anastomosis determines an advantage in terms of recovery outcomes and complications rate.

## Limitations

The major limitation of our study is its retrospective design and the small sample size which do not allow us to draw final conclusions; moreover, the multicentric enrollment which lasted 10 years does not permit to exclude a patients’ selection bias, particularly since different devices were used. Finally, because of a lack of consensus on the definition and assessment of post-surgical infections, results about this aspect must be read with caution.

Therefore, well-designed prospective multicentric trials and RCTs with homogeneous parameters are needed to set a real “gold standard” surgical procedure.

## Data Availability

Upon request to the corresponding author.
